# Multi-omics profiling reveals peripheral blood biomarkers of multiple sclerosis: implications for diagnosis and stratification

**DOI:** 10.3389/fphar.2024.1458046

**Published:** 2024-08-27

**Authors:** Qinming Zhou, Zuoquan Xie, Lu He, Guangqiang Sun, Huanyu Meng, Zhiyu Luo, Yuan Feng, Xingkun Chu, Liang Li, Jing Zhang, Yong Hao, Meiyu Geng, Xiang Zhang, Sheng Chen

**Affiliations:** ^1^ Department of Neurology and Institute of Neurology, Ruijin Hospital, Shanghai Jiao Tong University School of Medicine, Shanghai, China; ^2^ Co-innovation Center of Neuroregeneration, Nantong University, Nantong, China; ^3^ Clinical Center for Rare Diseases, Ruijin Hospital, Shanghai Jiao Tong University School of Medicine, Shanghai, China; ^4^ State Key Laboratory of Drug Research, Shanghai Institute of Materia Medica, Chinese Academy of Sciences, Shanghai, China; ^5^ Shanghai Green Valley Pharmaceutical Co., Ltd, Shanghai, China; ^6^ Departement of Neurology, Renji Hospital, Shanghai Jiao Tong University School of Medicine, Shanghai, China; ^7^ Shandong Laboratory of Yantai Drug Discovery, Bohai Rim Advanced Research Institute for Drug Discovery, Yantai, Shandong, China; ^8^ Department of Neurology, Huashan Hospital Fudan University and Institute of Neurology, Fudan University, Shanghai, China; ^9^ National Center for Neurological Disorders, Shanghai, China

**Keywords:** multiple sclerosis, multi-omics, blood immune phenotyping, proteomic, metabolomic, clinically isolated syndrome, secondary progressive MS

## Abstract

**Background:**

Multiple sclerosis (MS), a chronic autoimmune disorder marked by demyelination in the central nervous system, is exceptionally uncommon in China, and remains poorly understood in terms of its peripheral blood manifestations.

**Methods:**

We conducted a cohort study comprising 39 MS patients and 40 normal controls (NC). High-dimensional mass cytometry, protein arrays, and targeted metabolomics were utilized to profile immune subsets, proteins, and metabolites in blood. Differences in multi-omics signatures were scrutinized across varying MS subtypes.

**Results:**

Immune profiling demonstrated an elevation in various B cell subsets and monocytes, alongside a reduction in dendritic cells among MS patients. Proteomic data revealed a downregulation in neurotrophic and tissue repair proteins. Metabolomic assessment showed a noted decrease in anti-inflammatory molecules and sphingolipids. Integrated analysis identified distinct molecular patterns distinguishing MS from controls. Additionally, multi-omics differences among different MS subtypes were uncovered. Notably, hippuric acid levels was consistently lower in MS subgroups with greater disease severity.

**Conclusion:**

This study represents the pioneering exploration of multi-omics in Chinese MS patients, presenting a comprehensive view of the peripheral blood changes in MS. Our study underscores the robust capability of multi-omics assessments in identifying peripheral blood biomarkers that delineate the varied clinical presentation, and facilitates future development of biomarkers and targeted therapeutic interventions in MS.

## 1 Introduction

Multiple sclerosis (MS) stands as a persistent autoimmune affliction of the central nervous system, persisting without an established etiology. Within China, MS is considered a relatively uncommon condition, with the age- and sex-adjusted incidence of 0.235 per 100,000 person-years (Tian et al., 2020; Zhou et al., 2022). MS is characterized by a wide range of clinical presentations and an erratic progression, complicating the establishment of therapeutic strategies and prognostic assessments. Although significant strides have been made in molecular research, the exact processes that drive the advancement of MS are still not fully understood. Although there have been some indicators to monitor disease progression, a definitive clinical indicator is yet to be developed (Zhang et al., 2021; Zhou et al., 2022). This heterogeneity in clinical manifestations hinders both the prediction of disease outcomes and the formulation of treatment plans.

In the realm of medical practice, most MS cases initially manifest as clinically isolated syndrome (CIS), where symptoms suggestive of MS arise but a conclusive diagnosis often remains elusive (Koubiyr et al., 2020). While relapsing-remitting MS (RRMS) represents the most frequent clinical subtype of MS, the lack of effective control over relapses can result in patients advancing to secondary progressive MS (SPMS), for which treatments are notably scarce (Du et al., 2023). Nevertheless, clear-cut distinctions among CIS, RRMS, and SPMS have yet to be definitively delineated.

Over the past 10 years, “omics” methodologies have emerged as a powerful means for quantifying differentially expressed molecular entities. Their integration into MS research is anticipated to deepen our comprehension of the disease. To date, most omics investigations into MS have concentrated on cerebrospinal fluid (CSF) studies (Bhargava and Calabresi, 2016; Lorefice et al., 2023). While CSF may offer greater tissue specificity, obtaining a sample requires a lumbar puncture, which is uncomfortable and invasive. Moreover, prior researches into MS omics have primarily leveraged single omics methods, lacking an integrated understanding of the complex attributes of the disease (Singh et al., 2019c; Reinke et al., 2014). Plasma presents a compelling alternative due to its more accessible sampling and considerable promise in the identification of biomarkers that encapsulate the global expression profile from multiple tissues and cell types.

**FIGURE 1:**
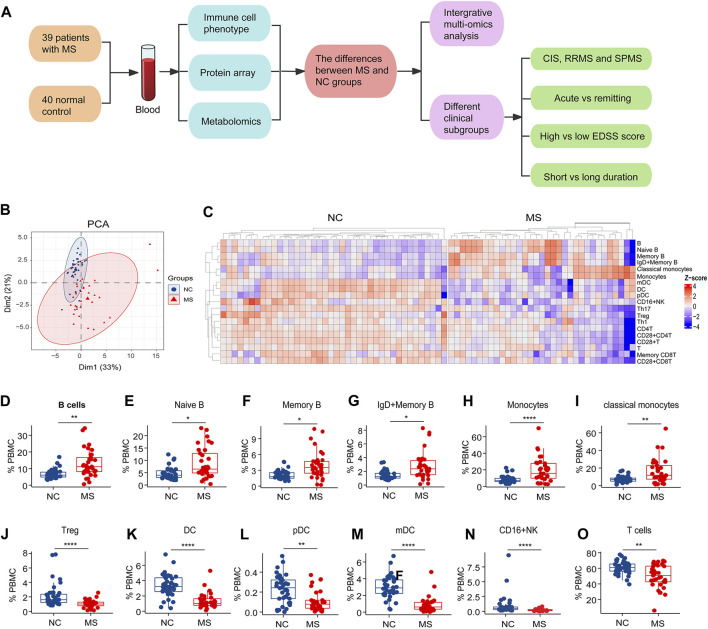
The study design and changes of blood immune subsets in MS. **(A)** The study flowchart. **(B)** PCA analysis of immune cells subsets between NC and MS. **(C)** Heatmap of differential expressed immune cell subsets (*p* < 0.05) in two groups (NC and MS). **(D–O)** Box plot of significantly differential immune cell subsets in MS compared to NC. ^*^ *P* < 0.05, ^**^ *P* < 0.01, ^***^ *P* < 0.001, ^****^ *P* < 0.0001, using Wilcox test.

In this study, we presented the pioneering exploration of multi-omics in Chinese patients with MS (Figure 1A). We employed a multi-omics framework, analyzing immune cell phenotypes, and levels of proteins and metabolites in peripheral blood samples from MS patients and normal controls (NC). And integrative analysis was used to identify peripheral signatures discriminate MS from NC. Comparative analyses were used differing MS clinical subtypes to identify markers reflective of disease activity and progression. The identified biomarkers bear potential for improving diagnostic accuracy, framing prognosis, and informing therapeutic approaches for individuals afflicted with MS.

## 2 Materials and methods

### 2.1 Participants

A cohort of 39 MS patients and 40 NC were recruited from Ruijin Hospital and Huashan Hospital, Shanghai, China. The study was approved by the Ethics Committee of Ruijin Hospital and Huashan Hospital and conducted in accordance with the principles of the Helsinki Declaration. All participants provided written informed consent.

All the MS patient fulfilled the international consensus diagnostic criteria for MS (Thompson et al., 2018). CIS is defined as a monophasic clinical episode characterized by patient-reported symptoms and objective findings that reflect a focal or multifocal inflammatory demyelinating event in CNS. This event develops acutely or subacutely, lasting for a minimum of 24 h, with or without recovery, and occurs in the absence of fever or infection. It is comparable to a typical multiple sclerosis relapse but occurs in a patient not known to have multiple sclerosis. In addition, all enrolled patients with CIS tested negative for oligoclonal bands. SPMS has a progressive course following an initial relapsing-remitting course (Oh et al., 2019). The progressive course is characterized by steadily increasing objectively documented neurological disability independent of relapses. The exclusion criteria were: 1) cardiovascular and/or metabolic diseases; 2) psychiatric disorders and/or neurologic disease other than MS; 3) body mass index (weight/height^2^) higher than 30; or 4) pregnancy.

### 2.2 Mass cytometry analysis of blood immune cells

Blood samples were collected from both MS patients and NC individuals in K2 EDTA tubes provided by BD (Part #366643). Post-centrifugation at 350 *g* for a duration of 10 min, the plasma obtained from the upper fraction was stored at −80°C for later analysis. The cell fraction at the bottom underwent lysis of red blood cells for 10 min at ambient temperature. These cells were subsequently rinsed twice with phosphate-buffered saline (PBS) and labeled using the Human Immune Monitoring Panel Kit from Fluidigm (Catalogue #201324). Initial staining with Cisplatin from Fluidigm (Catalogue #201064) was conducted at a concentration of 0.1 μM, followed by surface marker staining for 4 min. Fc receptors were blocked by a 10-min incubation with Cell Staining Buffer at room temperature. A blend of surface antibodies was introduced to the cells for half an hour while kept on ice. The cells were then washed using the staining buffer and fixed using paraformaldehyde (1.6% solution, Thermo Fisher, Catalogue #28908) for 10 min at ambient temperature. Cells were resuspended in Ir-Interchelator solution from Fluidigm (Catalogue #201192B) within Fix/Perm buffer (Fluidigm, Catalogue #201067) and incubated overnight within a temperature range of 2°C–8°C. Subsequently, cells were resuspended in the Fluidigm Cell Acquisition Solution (Catalogue #201237) amended with EQ Four Element Calibration beads at a 1:10 ratio (Fluidigm, Catalogue #201078) and passed through a 35 μm nylon mesh filtration cap (Corning, No. #352235). Acquisition of the cells was performed on a Helios Mass Cytometer (Fluidigm) at a collection rate of 200–300 events per second. Data from the mass cytometry were then transferred and processed using the Cytobank analysis platform, employing CD45highCD66-gating to remove granulocytes from the analysis.

### 2.3 Human Cytokine Antibody Array analysis

Protein detection in the plasma samples was performed by RayBiotech (Guangzhou, China), utilizing the Quantibody^®^ Human Cytokine Antibody Array 440 kit (RayBiotech, Inc., Catalog # QAH-CAA-440). The protocol was conducted in accordance with the guidelines provided by the manufacturer. Concisely, slide wells were prepped by adding 100 μL of sample diluent to each and allowing a 30-min incubation at ambient temperature to block non-specific binding sites. Subsequently, 100 μL of either the plasma samples or standard proteins were dispensed into the wells and permitted to incubate for 2 h at room temperature. The wells were then subjected to a series of washes—five times with Wash Buffer I and twice with Wash Buffer II, using 150 μL of each buffer for 5 min per wash, all at room temperature. Following the washes, each well received 80 μL of a detection antibody cocktail for a 2-h incubation at room temperature. Post-washing, wells were incubated with 80 μL of Cy3-equivalent dye labeled streptavidin for 1 h at room temperature. Following a final wash, the signal was captured via a laser scanning device.

### 2.4 MxP^®^ Quant 500 kit metabolite measurements

Targeted metabolomic profiling was conducted utilizing the MxP^®^ Quant 500 kit by BIOCRATES Life Sciences AG, Innsbruck, Austria, in conjunction with an advanced ultra-performance liquid chromatography/tandem mass spectrometry (UPLC-MS/MS) system comprising an ExionLC UPLC (Sciex) coupled with a QTRAP 6500+ triple quadrupole/linear ion trap MS/MS (Sciex) (Xie et al., 2024). This integrated system enables the quantitative and/or semiquantitative assessment of up to 630 endogenous and microbiota-related metabolites. Aligned with regulatory benchmarks set forth by the United States Food and Drug Administration (FDA) and the European Medicines Agency (EMA) for bioanalytical method validation, the MxP^®^ Quant 500 kit is specifically tailored and verified for use in human plasma analysis, incorporating internal and calibration standards to guarantee accurate quantification and consistent reproducibility within the analytical results.

### 2.5 Statistical analysis

To prepare the data for subsequent analysis, we excluded blood-borne immune cells, plasma cytokines, and metabolites with ≥30% missing values, followed by K-nearest neighbor (KNN) imputation of missing values.

The samples were from two cohorts: NC and MS. PCA (principal component analysis) is an unconstrained ordination method for dimension reduction. PCA could determinate PCs (principal component) which explain the most variance for data. Therefore, PCA projects high dimensional data on 2-dimensional scatterplot which enable the assessment of sample grouping. We used Wilcox to assess the associations of blood-borne immune cells, plasma, and cytokines with MS. We selected differential features based on Benjamini Hochberg adjusted *p*-values, with *p*-values <0.05 considered statistically significant. Heatmap was used to visualize the differential features profiles with ComplexHeatmap R package. Bar plots were made to visualize the change of differential features between MS and NC, CIS, RRMS and SPMS, acute phase and remitting phase, EDSS-H and EDSS-L, T2 and T1 by ggpubr R package.

### 2.6 Integrative multi-omics analysis

To identify important signatures that highly correlated among multi-omics and potentially discriminative for MS and NC, we performed Data Integration Analysis for Biomarker discovery using Latent Component (DIABLO) on omics data of the immune cells, the plasma proteins and metabolites (LC-MS). DIABLO is based on PLS-DA and aims to integrate multi-omics data by maximizing covariance between all pairs of datasets (Singh et al., 2019a). Prior to DIABLO, multi-omics data was log transformed. As discrimination is prioritized, the design matrix was set to 0.1. DIABLO performed 10 times repeat of 10-fold cross validation by block.splsda and tune.block.splsda functions in mixomics, to tune successively model hyperparameters for a final model which minimize classification error rate.

The first 2 components from the final model are considered and demonstrated in scatterplot using plotIndiv function for samples and plotVar function for features. Clustering of samples by group (NC/MS) and features by omics dataset was assessed. The mixomics package also provides loadingplot function and cim function to reveal important features (selected by DIABLO) for each omics dataset and combined in a heatmap, respectively. Heatmap that computed by cim function added a dendrogram with hierarchical clustering (Euclidean distance and complete linkage). The model performance was visualized by ROC curve calculated by auroc function also in mixomics. Only the importance features selected in component 1 of each omics dataset were assessed. DIABLO circles plot displayed the relationships between multiple sets of variables (correlation cut-off: r = 0.5) from component 1 and 2 that measured across the samples, which is useful in understanding how different types of biological data interact or correlate with each other.

## 3 Results

### 3.1 Characteristics of participants

The study enrolled 40 normal controls (NC group, 13 males and 27 females) and 39 MS patients (MS group, 11 males and 28 females). The average age for NC and MS groups was 35.3 ± 14.2 and 35.0 ± 14.5 years, respectively. Based on disease progression, the MS patients were further categorized into CIS (n = 14), RRMS (n =17), and SPMS (n = 8) groups. Within the MS group, a total of 33 patients were subjected to a blood immune cell subset analysis, 28 patients had their plasma protein levels assessed, and 30 patients underwent a plasma metabolomics profile analysis. The MS group had an average EDSS score of 2.0 ± 1.2, with an average disease duration of 22.8 ± 24.4 months (Supplementary Table S1).

### 3.2 Alterations of blood immune cell subsets in MS

To meticulously detail the variations in blood immune cell subclasses associated with MS, our study employed mass spectrometry to quantify 27 distinct immune cell subtypes (Supplementary Figure S1A). PCA revealed distinctive patterns of blood immune subsets in MS compared to the NC group (Figure 1B). We subsequently performed a comparative analysis of immune cell subtypes between the MS and NC cohorts.

Notably, both the general B cell population and specific subsets—naïve B cells, memory B cells, as well as IgD + memory B cells—alongside monocytes and classical monocytes, were found in increased numbers in the MS group relative to the NC group (Figures 1C–I).

Conversely, our analysis revealed a significant decrease in the levels of regulatory T cells (Tregs), dendritic cells (DCs), specifically both plasmacytoid DCs (pDCs) and myeloid DCs (mDCs), as well as CD16^+^ natural killer (NK) cells, T cells, and various T cell subsets, including CD4^+^ T cells, Th1, Th17, memory CD8^+^ T cells, CD28^+^ T cells, CD28^+^ CD4^+^ T cells, and CD28^+^ CD8^+^ T cells, within the MS group when compared to NC (Figures 1J–O; Supplementary Figures S1B–H).

### 3.3 Alterations of plasma proteins in MS

**FIGURE 2:**
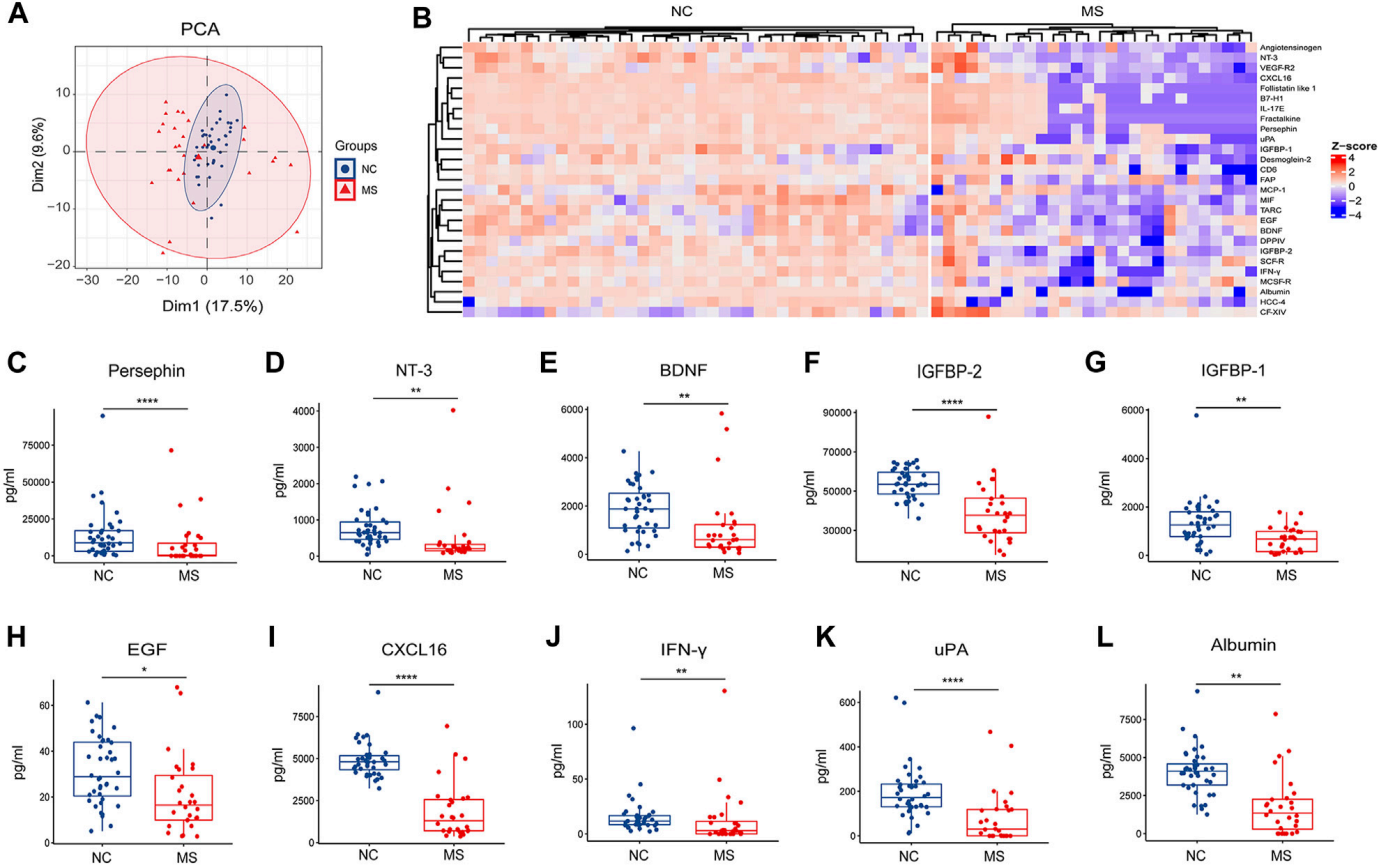
Changes of plasma proteins in MS. **(A)** PCA analysis of proteins between NC and MS. **(B)** Heatmap of differential proteins (*p* < 0.05) in two groups (NC and MS). **(C–L)** Box plot of significantly differential expressed plasma proteins in MS compared to NC. ^*^ *P* < 0.05, ^**^ *P* < 0.01, ^***^ *P* < 0.001, ^****^ *P* < 0.0001, using Wilcox test.

We initiated a protein array analysis targeting the expression levels of 440 plasma proteins. PCA analysis showed that the plasma protein expression patterns in MS were similar to those observed in the NC group (Figure 2A). Subsequent differential analysis revealed a broad spectrum of plasma proteins that were changed in MS (Figure 2B).

Notably, our findings demonstrate a significant reduction in the expression of neurotrophic factors within the MS group, specifically persephin, neurotrophin-3 (NT-3), and brain-derived neurotrophic factor (BDNF) (Figures 2C–E). In addition, there was a discernible decrease in the levels of insulin-like growth factor-binding protein 2 (IGFBP-2), insulin-like growth factor-binding protein 1 (IGFBP-1), and epidermal growth factor (EGF) in the MS group (Figures 2F–H). Some molecules linked with immune surveillance [C-X-C motif chemokine 16 (CXCL16) and interferon-gamma (IFN-γ)], urokinase-type plasminogen activator (uPA) and albumin were also found to be reduced in MS (Figures 2I–L).

### 3.4 Alterations of plasma metabolites in MS

**FIGURE 3:**
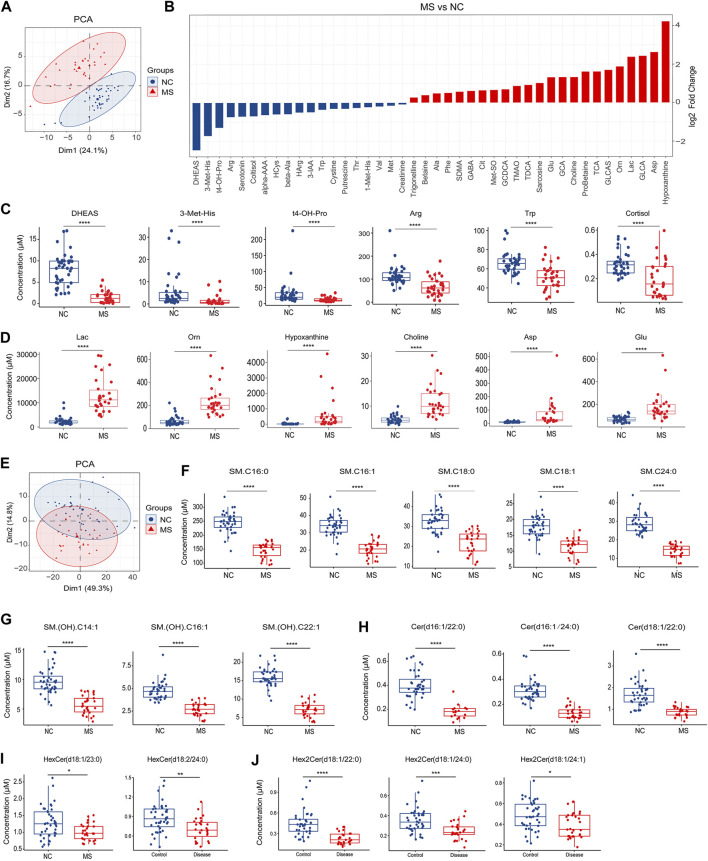
Changes of plasma metabolites in MS. **(A)** PCA analysis of plasma small molecular metabolites (LC-MS) in MS compared to NC. **(B)** Bar plot of significantly differential metabolites between NC and MS, *P* < 0.05. **(C)** Box plot of significantly decreased plasma metabolites in MS compared to NC. **(D)** Box plot of significantly increased plasma metabolites in MS compared to NC. **(E)** PCA analysis of plasma lipids (FIA) in MS compared to NC. **(F–J)** Box plot of significantly decreased plasma sphingolipids in MS compared to NC. ^*^ *P* < 0.05, ^**^ *P* < 0.01, ^***^ *P* < 0.001, ^****^ *P* < 0.0001, using Wilcox test.

Targeted metabolomics assessed the presence of 630 plasma metabolites to delineate differences between the MS and NC groups. We employed liquid chromatography-mass spectrometry (LC-MS) for the detection of small molecular metabolites and flow injection analysis (FIA) for large molecular lipids. The distinct profiles between MS and NC were observed in the PCA plots generated from LC-MS data (Figure 3A).

Comparative analysis of the small molecular metabolites uncovered numerous metabolites that were significantly altered in MS, (Figure 3B; Supplementary Figure S2A). Notably, the most dramatic declines for the MS group were observed for dehydroepiandrosterone sulfate (DHEAS), 3-methylhistidine (3-Met-His), trans-4-hydroxyproline (t4-OH-Pro), arginine (Arg), tryptophan (Trp), and cortisol (Figure 3C). In contrast, the most pronounced increases were found in lactate (Lac), ornithine (Orn), hypoxanthine, choline, aspartic acid (Asp), and glutamic acid (Glu) within the MS cohort (Figure 3D).

Regarding lipid metabolites, MS and NC groups were distinguishable as evidenced by the PCA plots (Figure 3E). Subsequent differential analysis between the MS and NC cohorts unmasked a multitude of lipid alterations (Supplementary Figure S2B). Crucially, our analysis underscored a pronounced reduction in sphingolipids within the MS group, including sphingomyelins (Figures 3F, G; Supplementary Figures S3A, B), ceramides (Figure 3H; Supplementary Figures S3C), hexosylceramides (Figure 3I), and dihexosylceramides (Figure 3J). Besides, we also identified a significant array of alterations in other lipid classes such as phosphatidylcholines (PCs), triglycerides (TGs), and cholesterol esters (CEs) (Supplementary Table S2).

### 3.5 Integrative features for discriminating MS from NC

**FIGURE 4:**
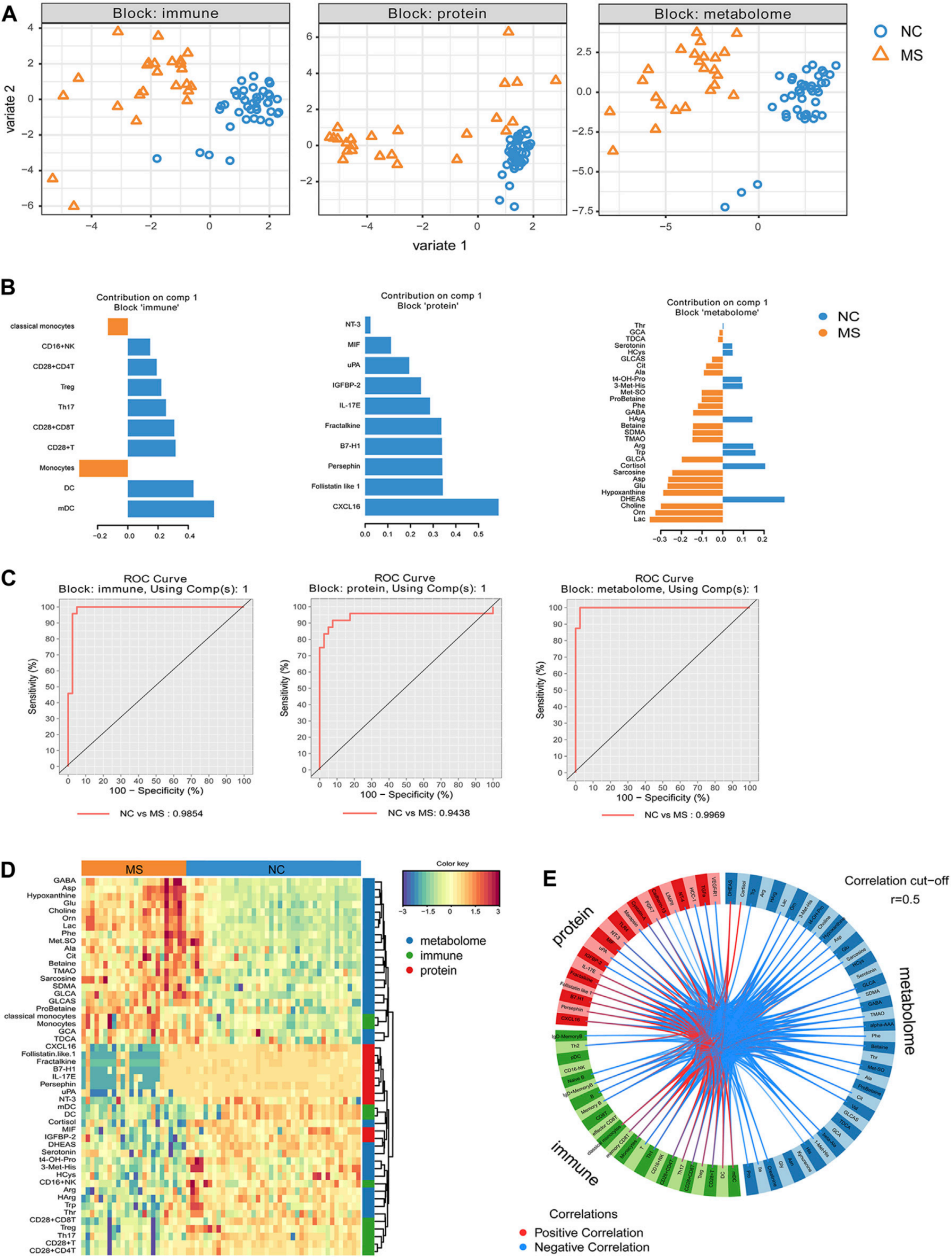
Integrative analysis revealed discriminative features between MS and NC. The highly correlated multi-omics signatures that discriminate NC and MS were identified from supervised model using DIABLO. **(A)** The scatterplot of samples using PlotIndiv on first 2 component for each block (immune cell subsets, proteins, small molecular metabolites). **(B)** Loading plot of component 1 from supervised model for each block, important signatures obtained from DIABLO were ordered by absolute importance (x-axis), colors indicated the class for which the median expression value is the highest for each feature. **(C)** ROC curve based on component 1 of each block (omics). ROC prediction models based on 70% of the samples and using 30% of the samples for prediction. **(D)** Heatmap of features selected by DIABLO. Dendrograms of features show the hierarchical relationship of the selected features based on Euclidean distance and complete linkage clustering. Samples on the x-axis are grouped by diagnosis (NC and MS). **(E)** DIABLO circle plot. Correlations between each feature from components 1 and 2 are plotted as a circle plot, and features with correlations above 0.5 (absolute value) were selected.

We conducted a DIABLO approach to identify a highly interrelated multi-omics signature that effectively differentiates MS from NC. Each aspect—immune subsets, proteins, and metabolites—displayed distinct profiles between MS and NC (Figure 4A). The immunity-related discrepancies consisted of augmented proportions of monocytes and classical monocytes in MS, in contrast to the higher proportions of DCs, myeloid dendritic cells (mDCs), and CD28^+^ T cells in NC. Within the proteomic landscape, NC demonstrated higher levels of CXCL16, follistatin-like 1, and persephin amongst other proteins; whilst metabolically, MS was characterized by higher levels of Lac, Orn, and choline, alongside diminished levels of DHEAS, cortisol, and Trp (Figure 4B). Employing these distinguishing features, we developed receiver operating characteristic (ROC) prediction models by training on 70% of the sample population and validating on the remaining 30%. This analysis demonstrated robust differentiation between MS and NC, with computed area under the curve (AUC) values of 0.98 for immune cell subtypes, 0.94 for proteins, and 0.99 for metabolites (Figure 4C).

Furthermore, DIABLO analysis revealed a pronounced correlation among monocytes, classical monocytes, and multiple metabolites (e.g., glycocholic acid [GCA], taurodeoxycholic acid [TDCA], glycolithocholic acid [GLCA], glycolithocholic acid sulfate [GLCAS], probetaine, symmetric dimethylarginine [SDMA], etc.), which were predominantly present in the MS group. There was also a tight correlation and lower representation of CXCL16, follistatin-like 1, fractalkine, programmed cell death 1 ligand 1 (B7-H1), interleukin-17E (IL-17E), Persephin, and uPA in MS. Additionally, another community featuring mDC, DC, cortisol, macrophage migration inhibitory factor (MIF), IGFBP-2, DHEAS, and others was closely correlated and underrepresented in MS (Figure 4D). Circus plot analysis further visualized the highly correlated variables (r > 0.5), showcasing the interconnectedness of these variables (Figure 4E).

### 3.6 Differences in peripheral signatures between different clinical subgroups

We categorized MS patients into distinct clinical subgroups based on various clinical parameters, such as disease stage, severity, phase, and disease duration. Our subsequent analysis aimed to elucidate differences in peripheral biomarkers among these diverse clinical subgroups.

**FIGURE 5:**
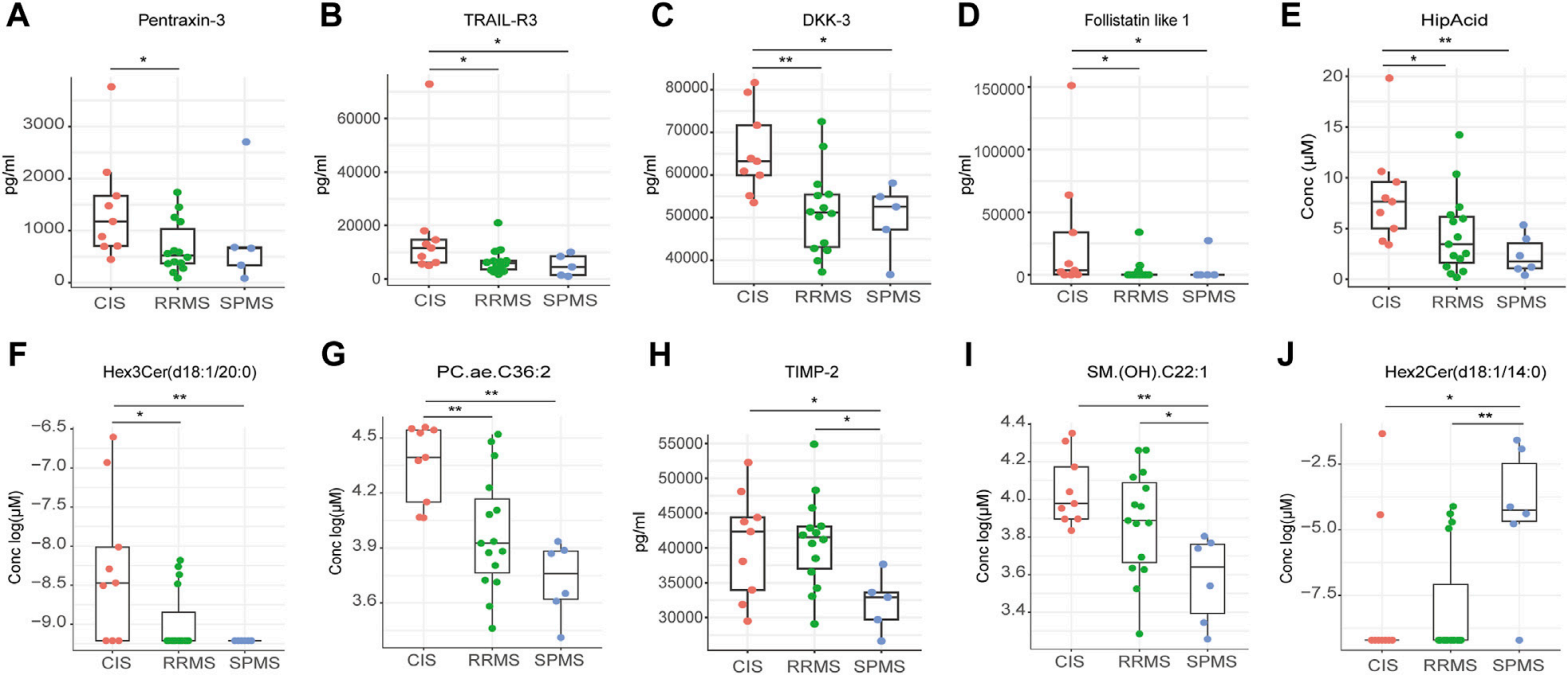
Differential peripheral features among CIS, RRMS, and SPMS. **(A–J)** Box plot showing the expression levels of proteins and metabolites among three groups. ^*^ *P* < 0.05, ^**^ *P* < 0.01, ^***^ *P* < 0.001, ^****^ *P* < 0.0001, using Kruskal-Wallis test.

Firstly, we assessed variances among the clinical stages of CIS, RRMS, and SPMS. Our investigation into immune cell population differences among these subgroups yielded no significant variances. However, notable differences emerged in protein and metabolites levels (Figures 5A–J; Supplementary Figures S4A–T). Notably, pentraxin-3 displayed a considerable reduction in RRMS compared to CIS, while tumor necrosis factor-related apoptosis-inducing ligand 3 (TRAIL-R3), dickkopf-3 (DKK-3), follistatin-like 1, hippuric acid (HipAcid), Hex3Cer(d18:1/20:0), and PC.ae.C36:2 exhibited decreased levels in both RRMS and SPMS relative to CIS (Figures 5A–G). Tissue inhibitors of metalloproteinases 2 (TIMP-2) and (SM) (OH).C22:1 were lower in SPMS compared to CIS or RRMS (Figures 5H, I). The level of Hex2Cer(d18:1/14:0) was higher in SPMS relative to CIS and RRMS (Figure 5J).

**FIGURE 6:**
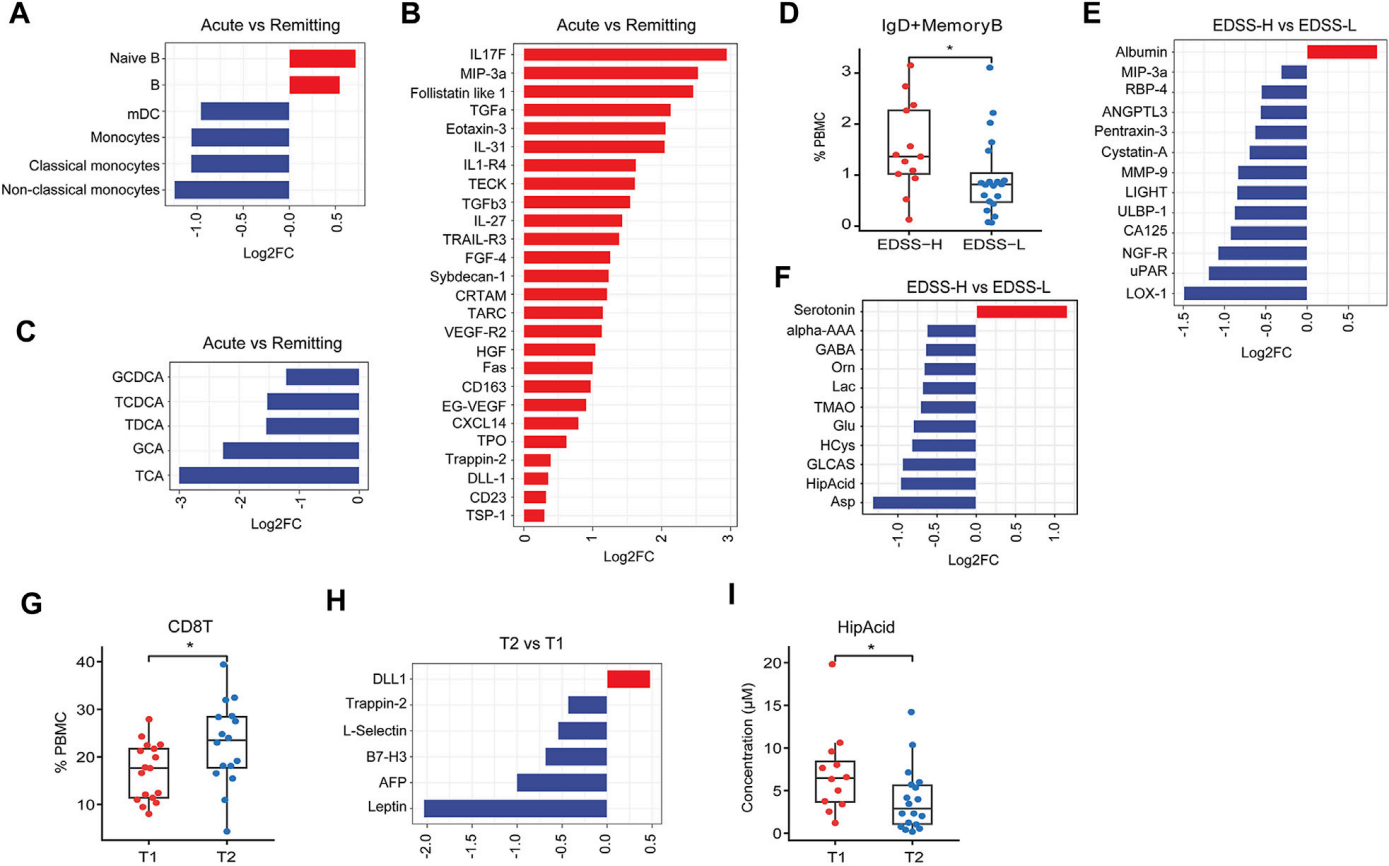
Difference in peripheral features between different clinical subgroups. **(A–C)** Bar plot of significantly differential expressed immune subset, proteins, metabolites (*P* < 0.05) between acute phase and remitting phase subgroups. **(D)** Box plot of IgD^+^ memory B cells in EDSS-H and EDSS-L subgroups. **(E, F)** Bar plot of significantly differential expressed proteins and metabolites (*P* < 0.05) between EDSS-H and EDSS-L subgroups. **(G)** Box plot of CD8T cells in T1 and T2 subgroups. **(H)** Bar plot of significantly differential expressed proteins (*P* < 0.05) between T2 and T1 subgroups. **(I)** Box plot of HipAcid in T1 and T2 subgroups. ^*^ *P* < 0.05, ^**^ *P* < 0.01, using Wilcox test.

Secondly, patients in the acute phase had a greater proportion of B cells and naïve B cells alongside lower levels of monocytes, classical and non-classical monocytes, and mDCs (Figure 6A). Concomitantly, the acute phase was associated with elevated concentrations of various proteins such as interleukin-17F (IL-17F), macrophage inflammatory protein-3 alpha (MIP3a), and follistatin-like 1 (Figure 6B). Additionally, analysis of the acute phase showed diminished levels of several bile acids [taurocholic acid (TCA), GCA, TDCA] relative to the remitting phase (Figure 6C).

Thirdly, we stratified patients according to the Expanded Disability Status Scale (EDSS). As compared to the EDSS-L group (EDSS<3), the EDSS-H group (EDSS ≥3) had an increased presence of IgD + memory B cells (Figure 6D), increased level of albumin and reduced levels of several proteins [lectin-like oxidized low-density lipoprotein receptor 1 (LOX-1), urokinase plasminogen activator receptor (uPAR), nerve growth factor receptor (NGF-R), etc.] (Figure 6E), the increased level of serotonin, and lower levels of several plasma metabolites (Asp, HipAcid, etc.) (Figure 6F).

Finally, MS patients were categorized based on disease duration into T1 (≤12 months) and T2 groups (>12 months). In comparison to T1 group, T2 group exhibited a higher ratio of CD8^+^ T cells (Figure 6G), lower concentrations of several proteins (leptin, AFP, B7-H3, etc.), higher level of delta-like ligand 1 (DLL1) (Figure 6H), and reduced levels of HipAcid (Figure 6I).

In addition, our study discerned substantial differential expression profiles of various lipid classes, including phospholipids, triglycerides, diglycerides, and sphingolipids, across distinct clinical subgroups of MS patients (Supplementary Figures S5A–C).

## 4 Discussion

In our current research, we conducted a comparative analysis of peripheral blood immune subsets, proteomic profiles, and metabolomic data between MS patients and healthy controls. This comparison identified distinct peripheral biomarkers that differentiate MS patients from NC and to examine the interrelationships among these biomarkers, as well as the peripheral difference in different subgroups of MS based on clinical classification.

### 4.1 Multi-omics offered a comprehensive depiction of the alterations in peripheral blood

In this study, substantial variations were detected in peripheral blood immune cell populations. Particularly noteworthy were the elevated levels of B cell and monocyte subsets, crucial for antibody synthesis and the mediation of inflammatory responses. Concurrently, Tregs, known for their immunosuppressive functions, were found to be depleted in MS, underscoring the central role of immune dysregulation in the disease’s pathogenesis. Additionally, a coordinated decline in DC and T cell subpopulations was identified in MS patients. These cell types are vital in immune surveillance, and their reduced numbers could increase the vulnerability to pathogens like viruses, which are potential etiological factors in MS(Tarlinton et al., 2020).

Proteomic analysis revealed the downregulation of several proteins associated with neural repair, including persephin, NT-3, BDNF, IGFBP-2, IGFBP-1, and EGF in MS patients. In the metabolomic profile, heightened levels of metabolites associated with inflammation or vascular injury, such as Lac, TMAO, choline, hypoxanthine, and cytotoxic bile acids, were noted. Concurrently, lower levels of anti-inflammatory metabolites such as DHEAS and cortisol (Rutkowski et al., 2014; Whitehouse, 2011) were observed. These findings collectively imply a role for peripheral inflammation and damage in the evolution of MS.

Interestingly, a significant decrease was observed in a few plasma sphingolipids in MS patients, including sphingomyelins, ceramides, and hexosylceramides. Since sphingolipids are key constituents of the myelin sheath and critical to CNS integrity and function, including cell growth, differentiation, and myelination (Hannun and Obeid, 2018; Leal et al., 2022; Dasgupta and Hogan, 2001), their decrement may influence remyelination processes. This result aligns with a prior study showing the decline of sphingolipids in the blood of MS patients (Momchilova et al., 2022). The underlying reasons for the decrease in these sphingolipids remain unclear; one possibility is increased clearance mediated by anti-sphingolipid antibodies. This hypothesis is in part supported by literature demonstrating elevated levels of antibodies against phosphatidylcholine in the serum of MS patients (Sádaba et al., 2020; Sánchez-Vera et al., 2023).

### 4.2 The distinctive features of peripheral blood changes across different clinical subtypes

To explore the difference in the pathogenesis among different clinical subtypes, we conducted comparisons of the immune subtypes, proteins, and metabolites. In the analysis comparing CIS, RRMS and SPMS, we found higher levels of TRAIL-R3, DKK-3, follistatin-like 1 in CIS than both RRMS and SPMS groups. TRAIL-R3 acts as a decoy receptor, mitigating apoptosis by sequestering the TRAIL ligand away from pro-apoptotic receptors (Jong et al., 2022), potentially aiding in cellular survival by hindering TRAIL-induced apoptotic pathways. DKK-3, a member of the Dickkopf protein family, regulates the Wnt signaling pathway, which influences cell fate determination, proliferation, and migration (Mourtada et al., 2023). Follistatin-like 1, known for its multifaceted role as a cytokine, exhibits a spectrum of functions from pro-inflammatory to anti-inflammatory actions (Mattiotti et al., 2018; Chaly et al., 2014). The observed decline in TRAIL-R3, DKK-3, and follistatin-like 1, HipAcid, Hex3Cer(d18:1/20:0) and PC.ae.C36:2 levels as MS progresses from CIS to RRMS or SPMS may hold implications for disease advancement.

In clinical practice, predicting the transition from RRMS to SPMS poses a significant challenge due to the absence of definitive biomarkers. Identifying early risk factors for SPMS development is crucial, as it allows for the timely initiation of effective treatment strategies aimed at mitigating disease progression. Our study revealed the significant reduction of TIMP-2 in SPMS patients compared to those with RRMS. TIMP-2, a member of the TIMP family, functions as an inhibitor of Matrix Metalloproteinase-2 (MMP-2). Previous studies showed that the presence of MMP-2 has been hypothesized to be linked to the chronic progressive phase of MS(Avolio et al., 2005; Fainardi et al., 2009). The interplay between TIMP-2 and MMP-2 is marked by TIMP-2’s irreversible inactivation of MMP-2 through its binding to the enzyme’s catalytic zinc cofactor. Moreover, Avolio et al. discovered that the serum MMP-2/TIMP-2 ratio was significantly higher in individuals with SPMS compared to those with RRMS(Avolio et al., 2003). This finding positions TIMP-2 as a promising candidate for a biomarker capable of distinguishing between SPMS and RRMS. Furthermore, the alterations observed in the levels of specific sphingolipids, such as Hex2Cer(d18:1/14:0) and SM. (OH).C22:1, in SPMS underscore the critical role these lipids may play in disease progression. The biological significance and repercussions of these specific variations are yet to be fully understood and thus represent a focal point for future research to clarify their impact on disease etiology and progression.

Furthermore, we investigated distinct characteristics across different levels of disability (EDSS-H vs EDSS-L), disease activity (acute vs remitting phase), and disease duration (T2 vs T1). Differential analysis between EDSS-H and EDSS-L groups revealed a higher abundance of IgD^+^ memory B cells in the EDSS-H group, suggesting an association with disease severity. Notably, albumin levels were significantly higher in the EDSS-H group. In the context of MS, the upsurge of albumin in circulation could stimulate the expression of pro-inflammatory cytokines and impair astrocytic function (LeVine, 2016). Consistent with previous literatures (Niino et al., 2009; Duddy et al., 2007; DiSano et al., 2021), our findings highlighted the elevated presence of B cells and naïve B cells in MS patients during the acute phase compared to the remission phase, reinforcing the pivotal role of B cells in MS pathogenesis. In contrast, both mDCs and monocyte levels were lower during the acute phase relative to the remission phase, possibly reflecting a depletion of innate immune cells during the acute phase, coinciding with a consistent elevation of plasma cytokines, indicative of an acute inflammatory response. Additionally, a higher representation of CD8^+^T cells was observed in patients with a more extended disease course, implying their involvement in long-term disease pathogenesis.

Our analysis revealed a noteworthy trend showing HipAcid levels to be consistently diminished in MS subgroups. The gradient observed when comparing CIS, RRMS, and SPMS, as well as among various subgroups (EDSS-H vs EDSS-L; acute vs. remitting; T2 vs T1), positions HipAcid as a compelling candidate biomarker for MS. HipAcid is a derivative of carboxylic acid produced in the liver through the enzymatic bonding of benzoic acid with glycine, and subjects with physical frailty generally exhibit reduced plasma and urine levels of HipAcid (Ticinesi et al., 2023). The noted reduction in HipAcid concentrations could be attributed to the frailty of greater severity subgroups.

Taken together, the discovery of substances potentially associated with the progression of MS may offer valuable clues for future research into the mechanisms of disease progression. This could aid in better identifying MS patients prone to progression, enabling early intervention, and slowing the progression of disability.

### 4.3 The integration analysis revealed a strong correlation among various categories of biological molecules

DIABLO is a multivariate analytical technique that integrate diverse categories of “omics” data to identify biomarkers with consistent significance across various layers (Singh et al., 2019b). In the context of our study, the DIABLO method facilitated the discernment of a clear distinction between MS patients and NC, enabling us to identify key peripheral signatures. A range of critical biomarkers emerged as top signatures for discriminating MS from NC, including mDC, DC, monocytes, CXCL16, follistatin-like 1, persephin, Lac, Orn, and choline, among others. Importantly, the study unveiled variables that were highly intercorrelated across three distinct “omics” analyses. The comprehensiveness of the DIABLO approach allowed for the construction of an in-depth profile that not only distinguishes MS from NC but also elucidates the complex interrelationships inherent within each group, affording novel insights into their respective biological processes.

## 5 Limitation

Firstly, given the relatively low prevalence of MS, the sample size included in our study was limited, specifically for patients with CIS or SPMS. Secondly, the research methodology employed was of a retrospective and cross-sectional nature, which inherently presents certain limitations in capturing the full longitudinal trajectory of the disease. Thirdly, the exclusivity of our analysis to MS without juxtaposition against other neurological disorders may limit the specificity of our findings to this disease. Lastly, the selected patients had been subjected to a variety of treatments, including no treatment, corticosteroids, and various disease-modifying therapies, making it difficult to rule out the potential interference on the results of the study.

## 6 Conclusion

This was the first study of multi-omics in Chinese MS patients, to elucidate the multi-faceted molecular alterations in the peripheral blood of MS patients. The study further distinguished multi-omics signatures among different MS clinical subtypes. The findings provided a nuanced view of the disease’s systemic impact, and underscored their relevance for pathogenetic studies, disease monitoring, and the early detection of progressive MS subtypes. Future work will focus on validating these findings in a larger cohort, and elucidating the mechanistic basis of these biomarkers with the goal of improving patient outcomes.

## Data Availability

The data analyzed in this study is subject to the following licenses/restrictions: The data that support the findings of this study are available from the corresponding author upon reasonable request. Requests to access these datasets should be directed to Sheng Chen, mztcs@163.com.
