# Supplementary material for: Multi-omics profiling reveals peripheral blood biomarkers of multiple sclerosis: implications for diagnosis and stratification
Source: Front Pharmacol. 2024 Aug 27;15:1458046. doi: 10.3389/fphar.2024.1458046 (PMC11384994; doi:10.3389/fphar.2024.1458046)
Supplement: Supplementary file 3 [file DataSheet1.docx]

**Supplementary Figures**


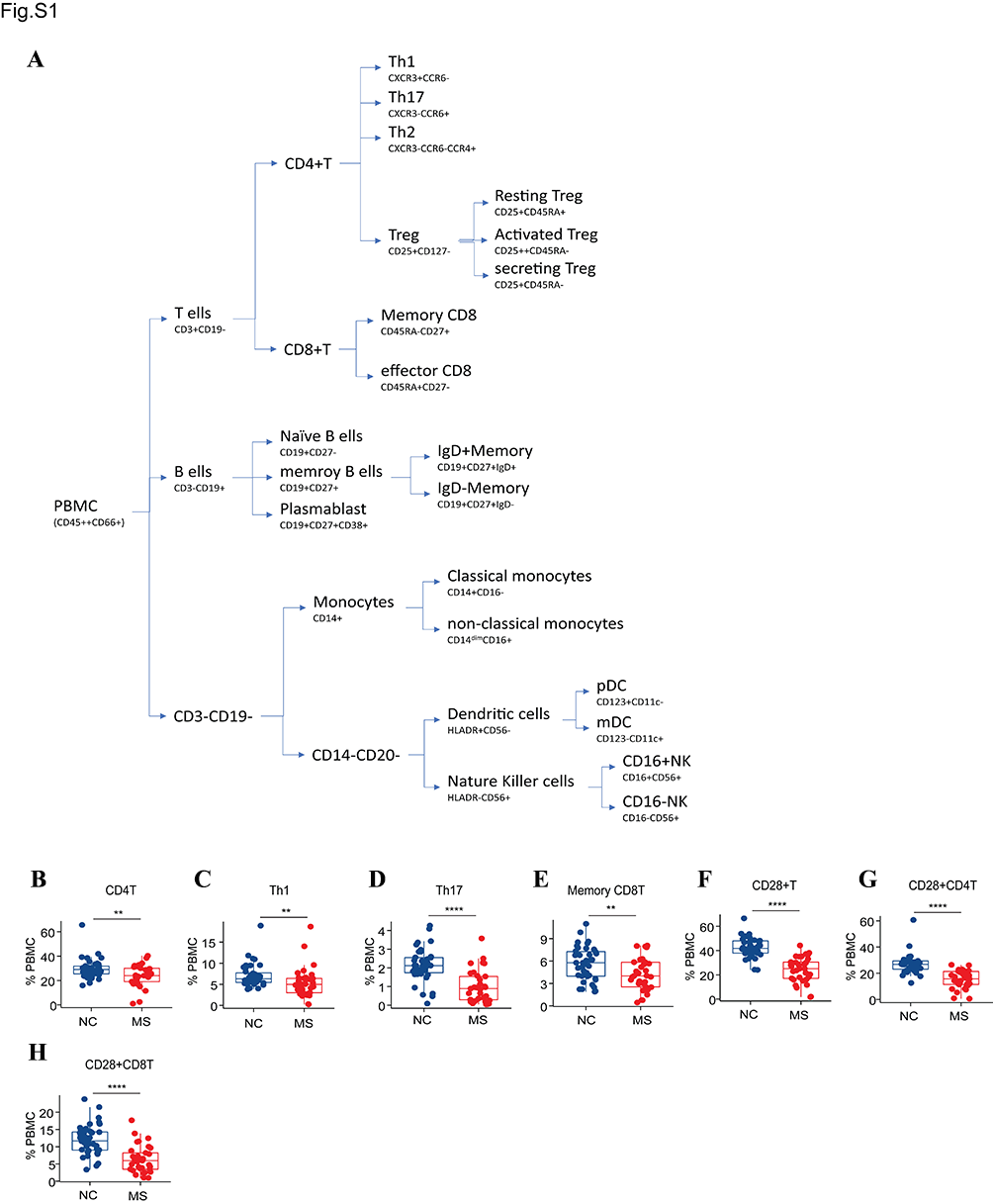


**Figure S1.** Changes of blood immune subsets in MS. (A) Flow chat of immune cell subtyping. (B-H) Bar plot of significantly differential immune cell subsets in MS compared to NC. ^*^*P*<0.05, ^**^*P*<0.01, ^***^*P*<0.001, ^****^*P*<0.0001, using Wilcox test.


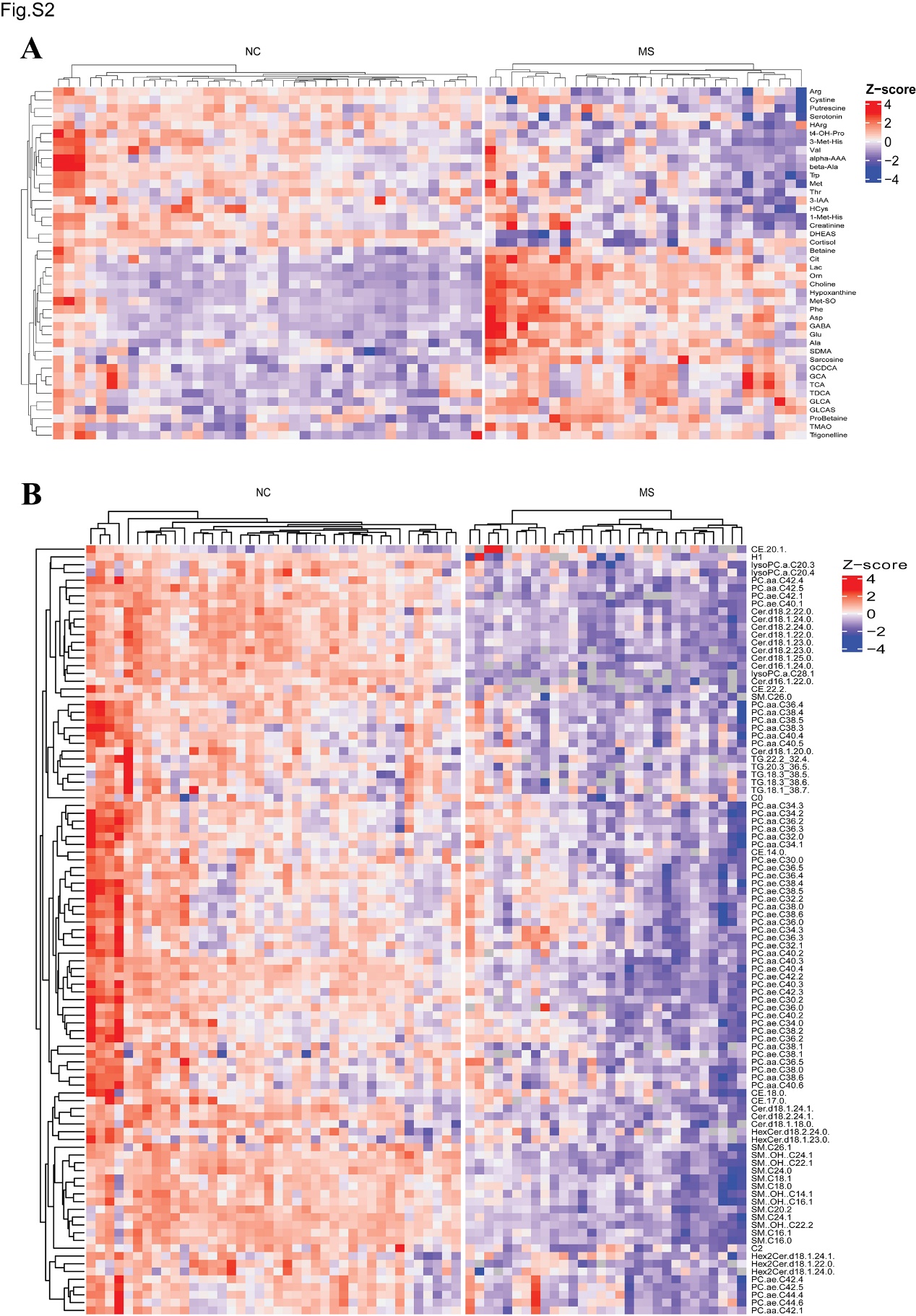


**Figure S2.** Differential metabolites in plasma of MS as compared to NC. (A) Heatmap of differential plasma small molecular metabolites (*P* < 0.05) between MS and NC, as determined by LC-MS. (B) Heatmap of differential plasma lipids (*P* < 0.05) between MS and NC, as determined by FIA.


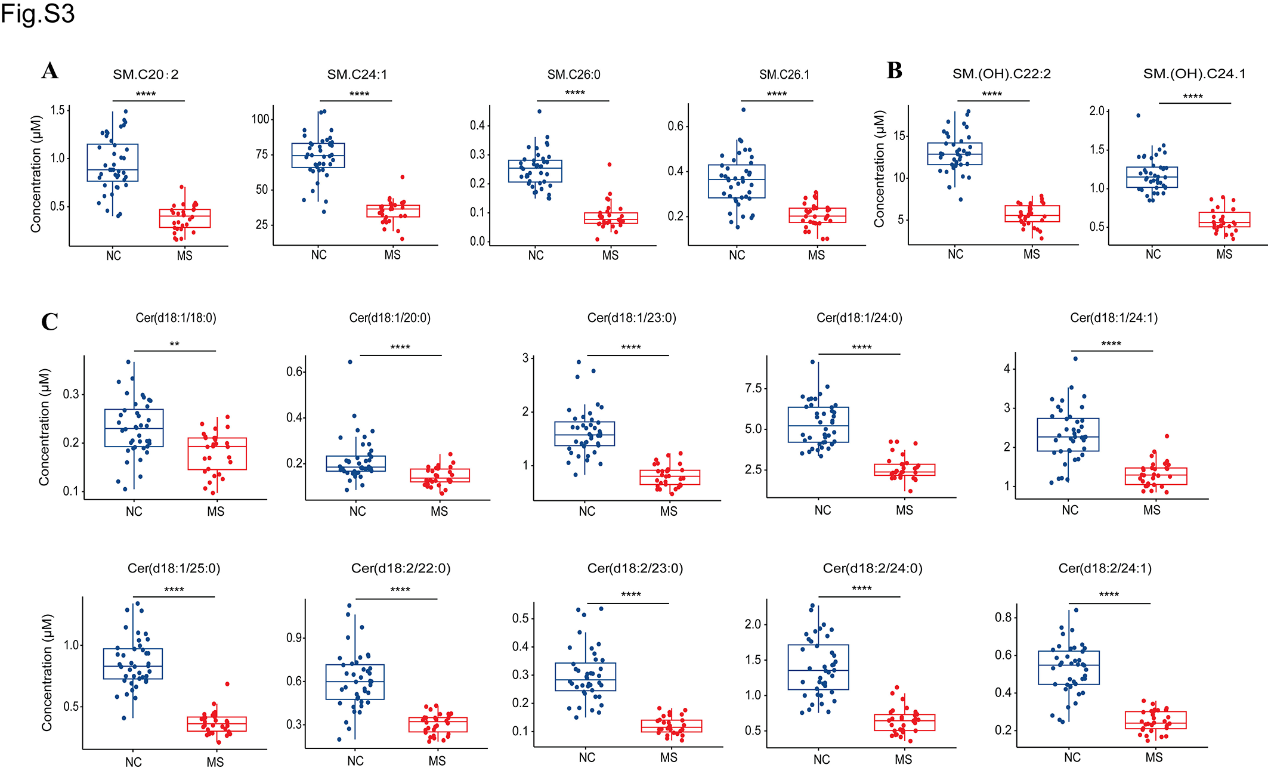


**Figure S3.** Changes of plasma lipids in MS. (A-C) Box plot of significantly changes plasma lipids in MS compared to NC. ^*^*P*<0.05, ^**^*P*<0.01, ^***^*P*<0.001, ^****^*P*<0.0001, using Wilcox test.


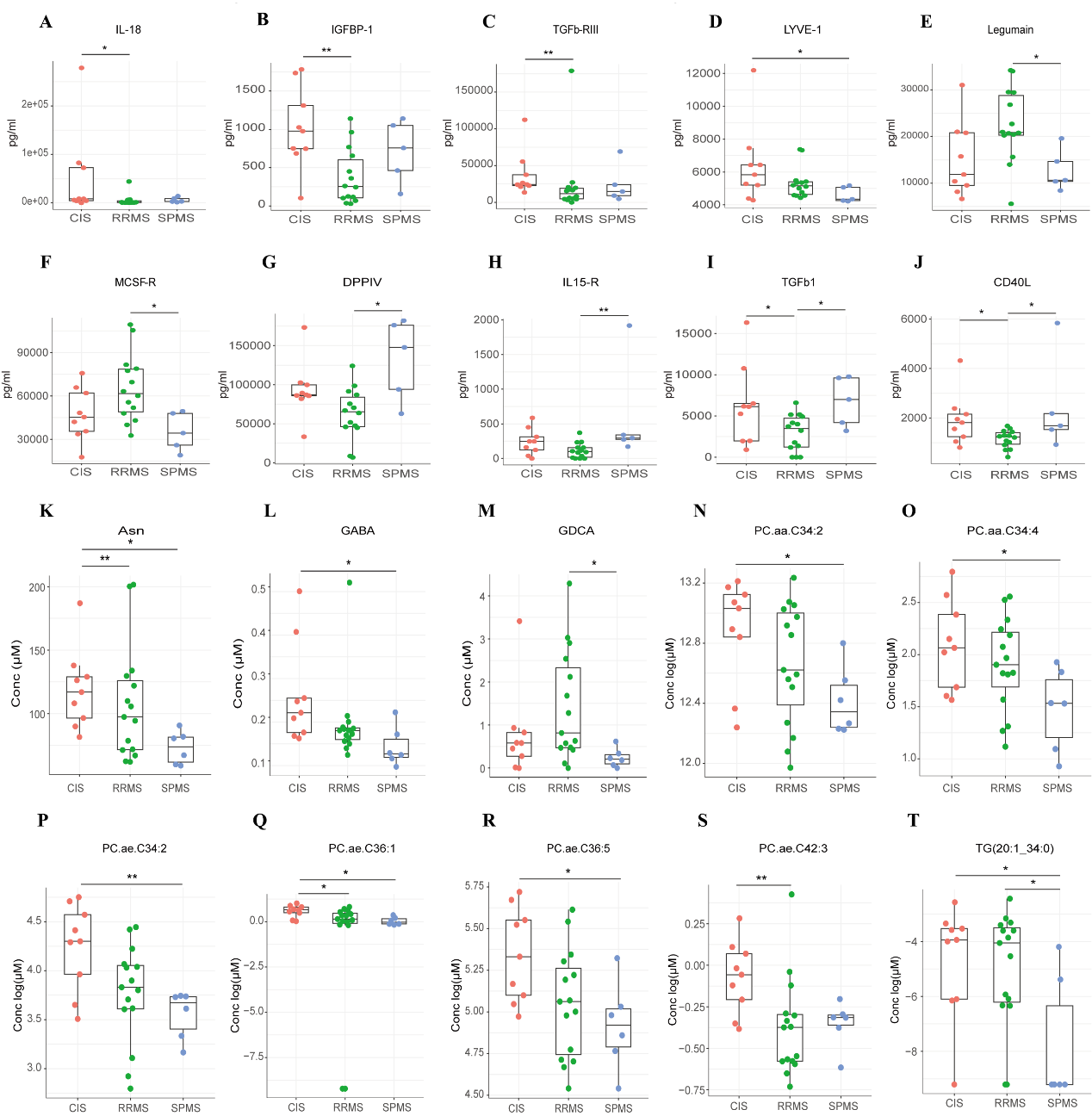


**Figure S4.** Differential proteins and metabolites among CIS, RRMS, and SPMS. (A-T) Box plot showing the expression levels of proteins and metabolites among three groups. ^*^*P*<0.05, ^**^*P*<0.01, ^***^*P*<0.001, ^****^*P*<0.0001, using Kruskal-Wallis test.


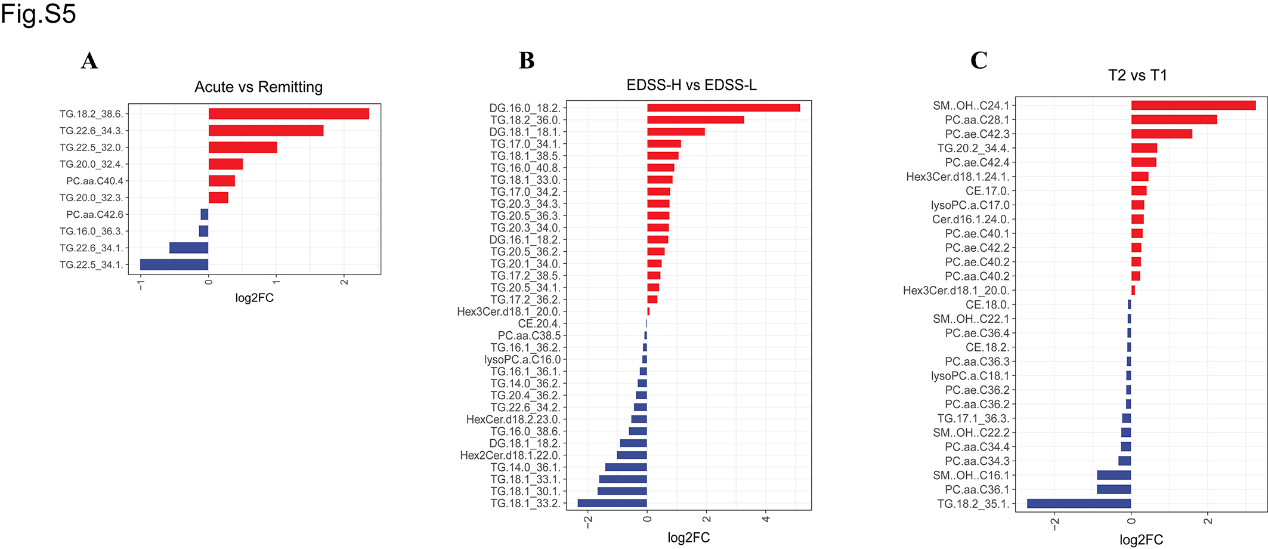


**Figure S5.** Difference in plasma lipids between different clinical subgroups. (A) Bar plot of significantly differential expressed lipids (*P*<0.05) between acute phase and remitting phase subgroups. (B) Bar plot of significantly differential expressed lipids (*P*<0.05) between EDSS-H and EDSS-L subgroups. (C) Bar plot of significantly differential expressed lipids (*P*<0.05) between T2 and T1 subgroups. *P*<0.05, using Wilcox test.
